# Supplementary material for: Trpm5 channels encode bistability of spinal motoneurons and ensure motor control of hindlimbs in mice
Source: Nat Commun. 2021 Nov 24;12:6815. doi: 10.1038/s41467-021-27113-x (PMC8613399; doi:10.1038/s41467-021-27113-x)
Supplement: Supplementary file 1 — Supplementary Information [file 41467_2021_27113_MOESM1_ESM.pdf]

# SUPPLEMENTARY INFORMATION

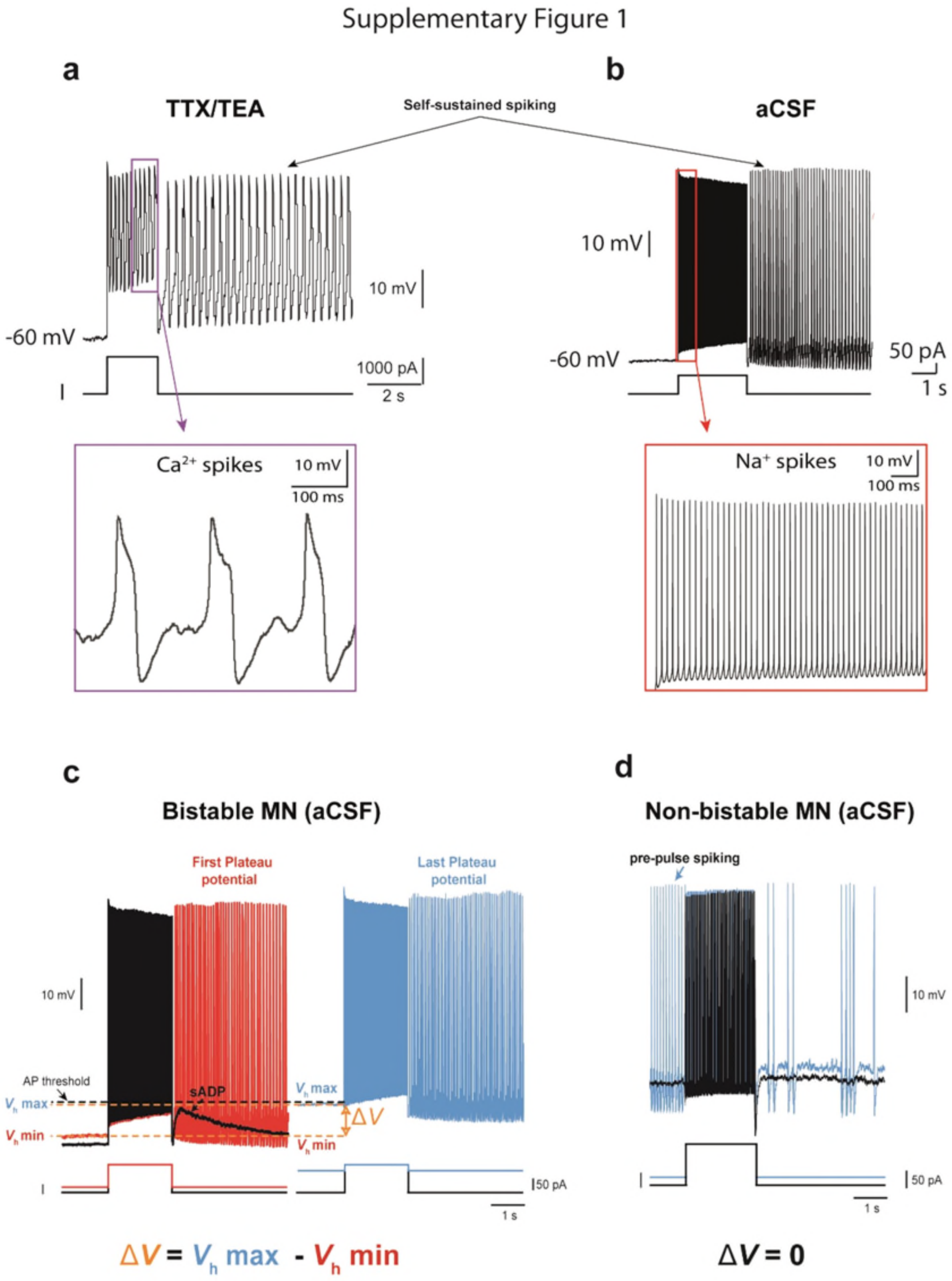


**Figure S1: Functional description of bistable parameters from ventro-lateral lumbar motoneurons (related to Fig. 1). a,b** Example of self-sustained spiking induced by a 2-s single pulse in normal TTX/TEA conditions (**a**) and in aCSF (**b**). In (**a**)

the cell firing is characterised by slow (~100 ms) and small amplitude calcium spikes (~ 30 mV) (purple inset) while in (**b**), the cell firing is characterised by fast (0.5 ms) and large amplitude sodium spikes (~60 mV) (red inset). **c** Bistable cell is characterized by its ability to stay (1) in a silent downstate during the pre-stimulus period for a range of holding potentials ($\Delta V$), and (2) in the firing up-state during the post-stimulus period. The $\Delta V$ is considered as the difference between the most hyperpolarized ($V_h$ min) and the most depolarized ($V_h$ max) holding potentials at which self-sustained spiking can be triggered. **d** Non-bistable cell is characterized by its inability to express a post-stimulus self-sustained firing before reaching spiking activity during pre-stimulus holding potential. Therefore $\Delta V$ is null.

Figure S2

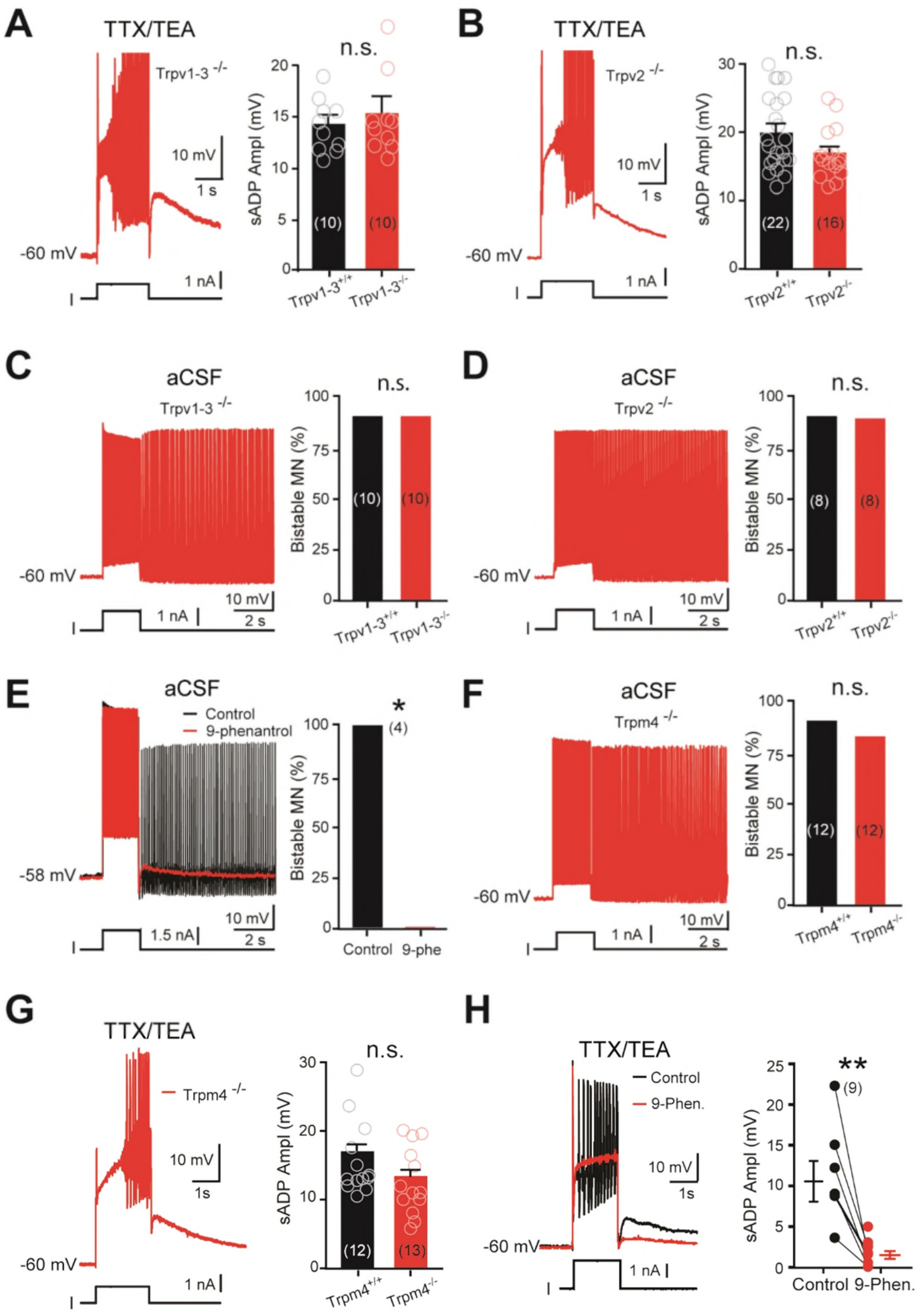
A
TTX/TEA
Trpv1-3 -/-
10 mV
1 s
-60 mV
1 nA
n.s.
sADP Ampl (mV)
(10)
(10)
Trpv1-3+/+
Trpv1-3-/-
B
TTX/TEA
Trpv2 -/-
10 mV
1 s
-60 mV
1 nA
n.s.
sADP Ampl (mV)
(22)
(16)
Trpv2+/+
Trpv2-/-
C
aCSF
Trpv1-3 -/-
-60 mV
1 nA
10 mV
2 s
n.s.
Bistable MN (%)
(10)
(10)
Trpv1-3+/+
Trpv1-3-/-
D
aCSF
Trpv2 -/-
-60 mV
1 nA
10 mV
2 s
n.s.
Bistable MN (%)
(8)
(8)
Trpv2+/+
Trpv2-/-
E
aCSF
Control
9-phenantrol
-58 mV
1.5 nA
10 mV
2 s
*
(4)
Bistable MN (%)
Control
9-phe
F
aCSF
Trpm4 -/-
-60 mV
1 nA
10 mV
2 s
n.s.
Bistable MN (%)
(12)
(12)
Trpm4+/+
Trpm4-/-
G
TTX/TEA
Trpm4 -/-
10 mV
1s
-60 mV
1 nA
n.s.
sADP Ampl (mV)
(12)
(13)
Trpm4+/+
Trpm4-/-
H
TTX/TEA
Control
9-Phen.
10 mV
1s
-60 mV
1 nA
**
(9)
sADP Ampl (mV)
Control
9-Phen.

**Supplementary Fig. 2 (related to Fig. 2): The $I_{CaN}$-mediated sADP and bistability of motoneurons are not dependent on Trpv1-3 and Trpm4 channels. a-h** Left: representative voltage traces of motoneurons recorded in response to a depolarizing pulse with (**a,b,g** and **h**) or without TTX/TEA (**c-f**) from wild-type mice before and after bath-applying 9-phenanthrol (**e** and **h**, 50 µM, *n* = 5 mice), or recorded in motoneurons from mutant mice lacking Trpv1-3 (**a** and **c**, *n* = 4 mice), Trpv2 (**b** and **d**, *n* = 5 mice) or Trpm4 (**f** and **g**, *n* = 6 mice) channels, right: group mean quantification of the peak amplitude of the sADP (**a,b,g** and **h**) or of the proportion of bistable motoneurons (**c-f**). Numbers in brackets indicate the numbers of recorded motoneurons. Each circle represents an individual motoneuron. n.s., no significance; *$P < 0.05$ (two-tailed Mann-Whitney test for **a,b** and **g**; two-tailed Fisher test for **c-f**; two-tailed Wilcoxon paired test for **h**). Mean ± SEM. For detailed *P* values see Source Data. Source data are provided as a Source Data file.

Supplementary Figure 3

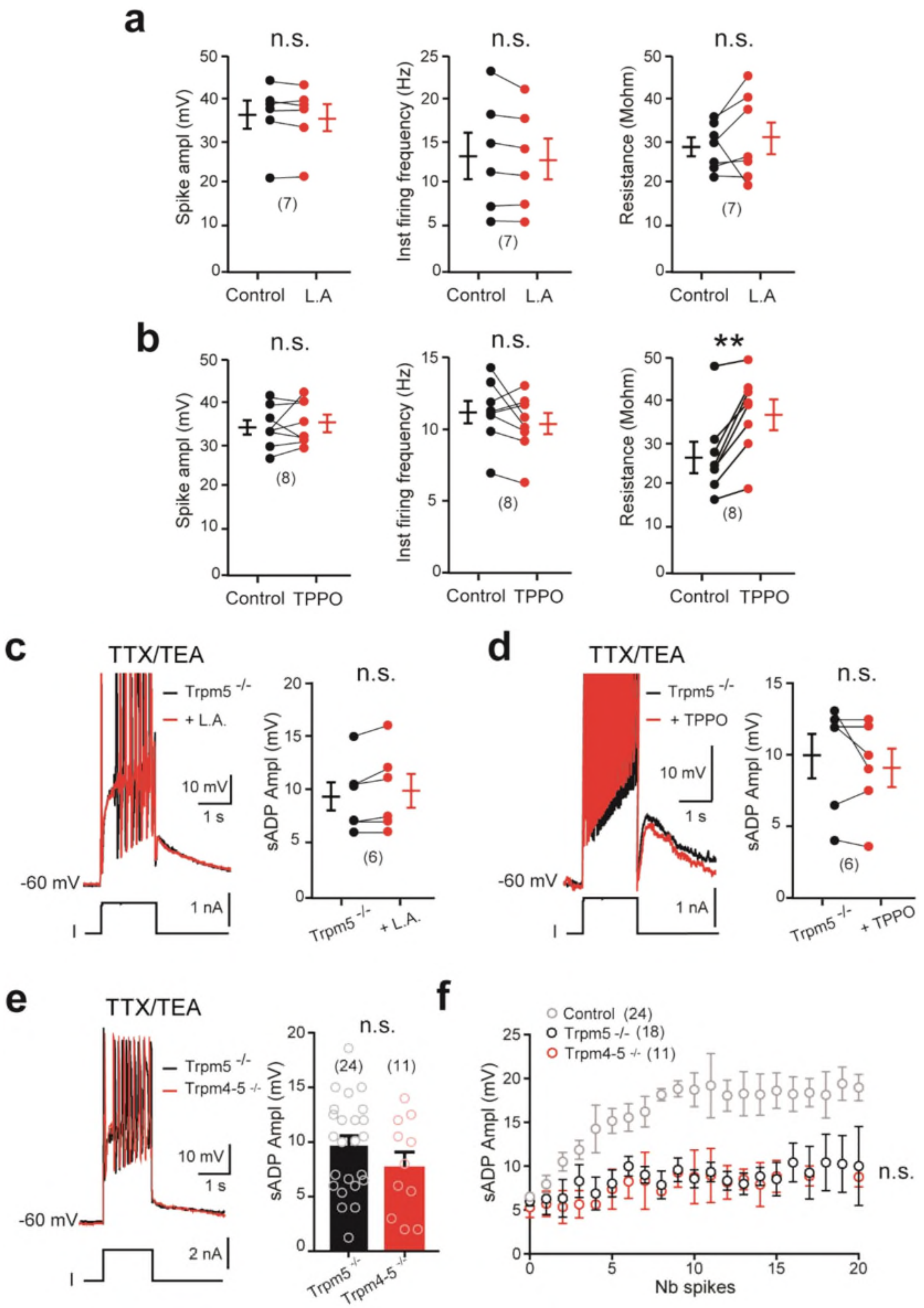


**Supplementary Fig. 3 (related to Fig. 2): Validation of linoleic acid and triphenylphosphine oxide as specific modulators of Trpm5 channels. a,b** Mean calcium spike amplitude (left), instantaneous spiking frequency (middle) and input

resistance (right) recorded from lumbar motoneurons under TTX/TEA in the absence (control) or the presence of Trpm5 modulators, Linoleic acid (L.A., 30-50 µM; *n* = 3 mice; **a**) or triphenylphosphine oxide, (TPPO, 30-50 µM; *n* = 4 mice; **b**). **c,d** Left: representative voltage traces of motoneurons in response to a depolarizing pulse recorded under TTX and TEA from Trpm5$^{-/-}$ mice in absence (black) or in presence (red) of L.A. (30 µM; **c**) or TPPO (50 µM; **d**), right: mean amplitude of the sADP (*n* = 4 mice). **e** Left: representative voltage traces of motoneurons in response to a depolarizing pulse recorded under TTX and TEA from Trpm5$^{-/-}$ mice (black trace) and from a double knock-out Trpm4-5$^{-/-}$ mice (red trace), right: mean amplitude of the sADP (*n* = 5 Trpm5$^{-/-}$ mice and *n* = 3 Trpm4-5$^{-/-}$ mice). **f** Relationship between the peak amplitude of the sADP and the number of spikes emerging during the 2-s depolarizing current pulse. Numbers in brackets indicate the numbers of recorded motoneurons. Each circle represents an individual motoneuron. (*n* = 9 control mice, *n* = 5 Trpm5$^{-/-}$ mice and *n* = 3 Trpm4-5$^{-/-}$ mice) n.s., no significance; $^{**}P < 0.01$ (two-tailed Wilcoxon paired test for **a-d**; two-tailed Mann-Whitney for **e**; one-way ANOVA with multiple comparisons for **f**). Mean ± SEM. For detailed *P* values see Source Data. Source data are provided as a Source Data file.

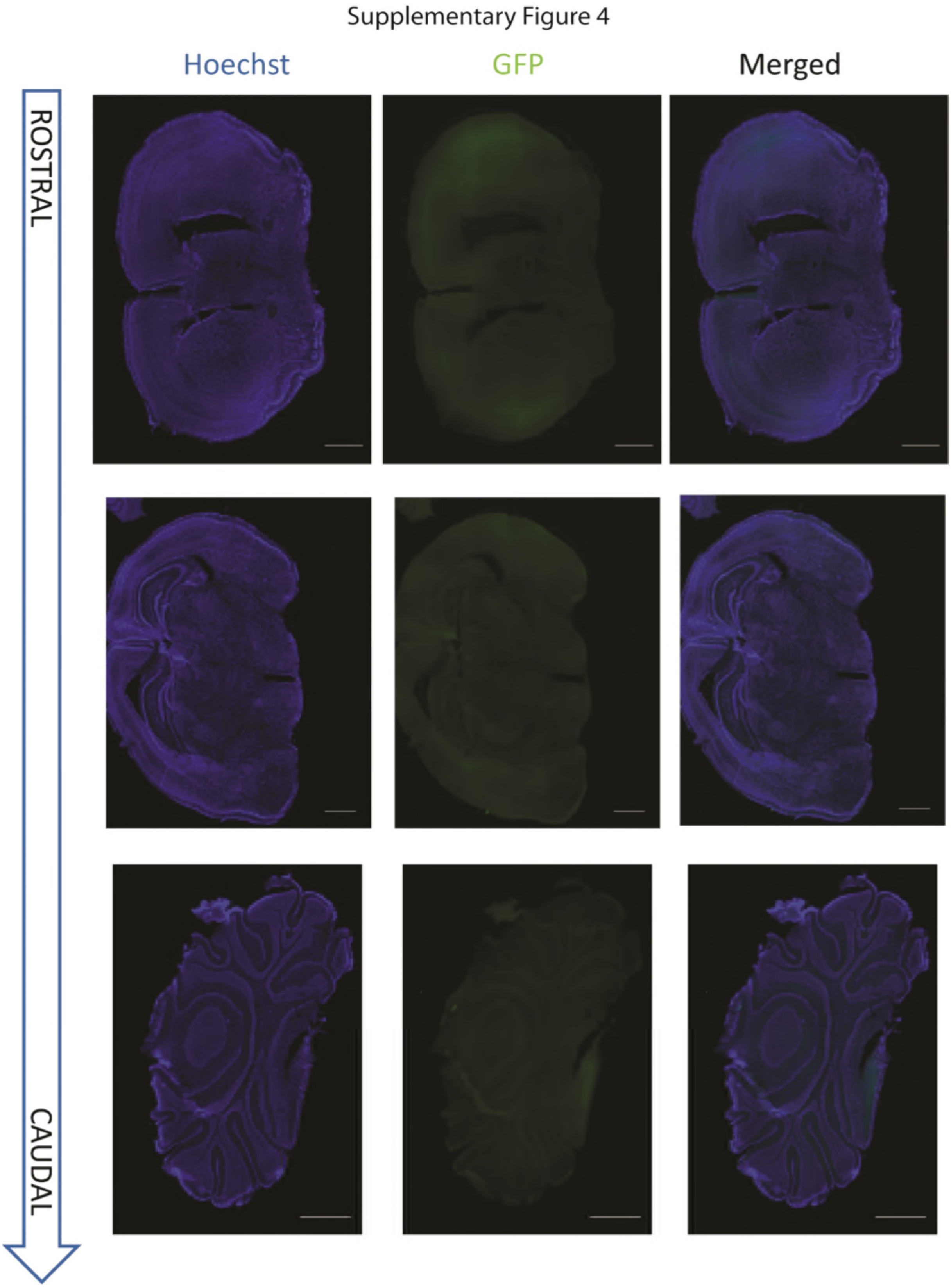


**Supplementary Fig. 4 (related to Fig. 2). Absence of brain transduction following spinal intrathecal AAV9 administration.** Representative confocal images of Hoechst

dye (left) and eGFP fluorescence signal (middle) across three different coronal sections of the brain from the same P10 mouse illustrated in Fig. 2e. The intrathecal administration of AAV9 harboring a self-complementary genome expressing CMV.eGFP and ShRNA-Trpm5 was performed at birth on 4 different mice. All 4 injected mice do not display any GFP fluorescence in the represented brain structures. Scale bar: 100 µM.

## Supplementary Figure 5

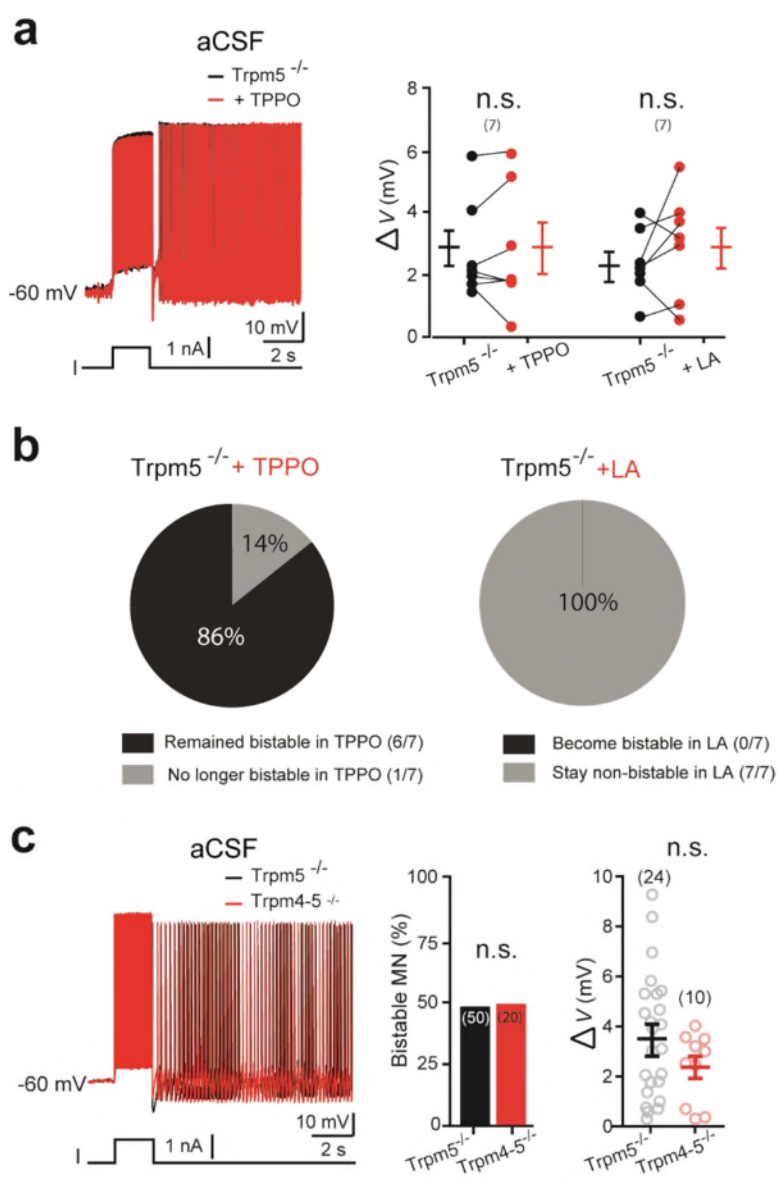


**Supplementary Fig. 5 (related to Fig. 3): Bistable properties recorded from Trpm5$^{-/-}$ mice are not affected by Trpm5 modulators and are similar to those recorded from Trpm4-Trpm5 double knock-out mice. a** Left: superimposed voltage traces recorded in motoneurons from Trpm5$^{-/-}$ mice before (black) and after (red) bath-applying the Trpm5 blocker triphenylphosphine oxide, (TPPO, 50 µM), right: mean quantification of the Δ*V* (*n* = 2 mice for each condition, TPPO and LA). Δ*V* was defined as the difference between spiking threshold and the most hyperpolarized holding

potential for which self-sustained firing can be triggered. **b** Group mean quantification of the proportion of bistable motoneurons recorded from Trpm5$^{-/-}$ mice under TPPO (left, *n* = 2 mice) or L.A. (right, *n* = 2 mice). **c** Left: superimposed voltage traces recorded in motoneurons from Trpm5$^{-/-}$ mice (black) and from a double knock-out Trpm4-5$^{-/-}$ mice (red). Group mean quantification of the proportion of bistable motoneurons from Trpm5$^{-/-}$ (black, *n* = 7 mice) and Trpm4-5$^{-/-}$ (red, *n* = 3 mice) mice and of $\Delta V$. Numbers in brackets indicate the numbers of recorded motoneurons. Each circle represents an individual motoneuron. n.s., no significance; [two-tailed Wilcoxon paired test for **a**; two-tailed Fisher test for **b** and **c** (middle); two-tailed Mann-Whitney test for **c** (right)]. Mean ± SEM. For detailed *P* values see Source Data. Source data are provided as a Source Data file.

## Supplementary Figure 6

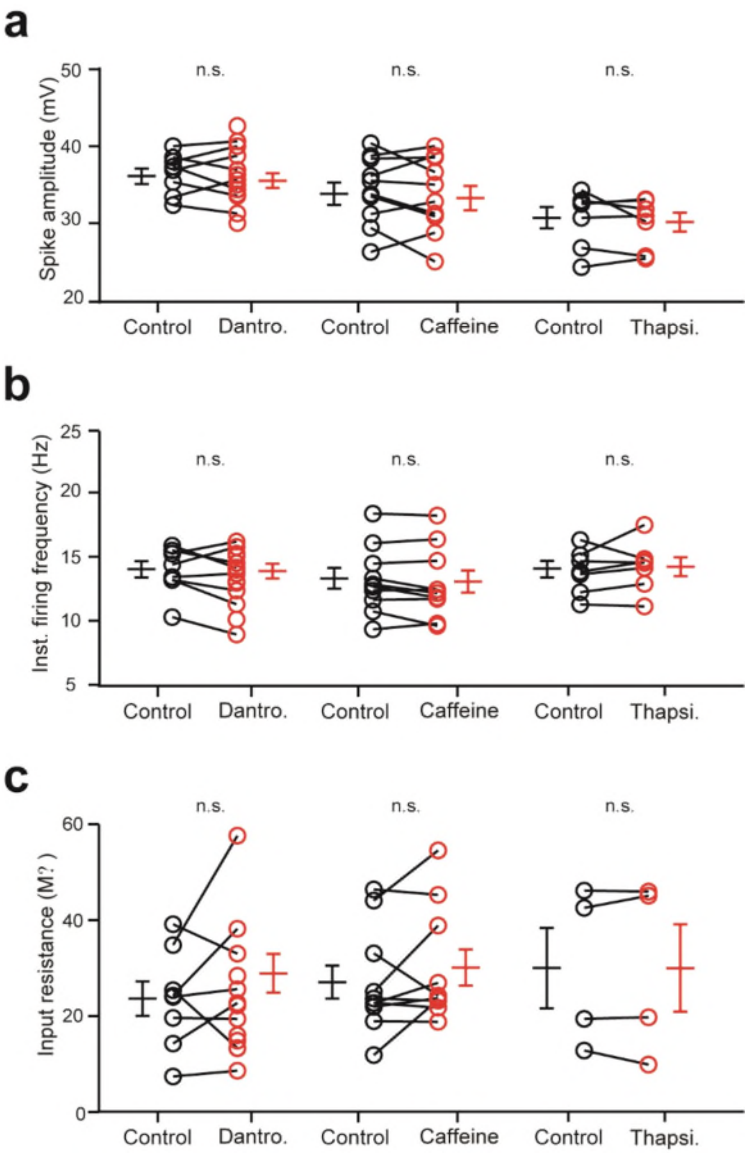


**Supplementary Fig. 6 (related to Fig. 4): Dantrolene, caffeine and thapsigargin do not alter $Ca^{2+}$ spiking activity. a-c** Mean calcium spike amplitude (**a**), instantaneous firing frequency (**b**) and input resistance (**c**) of motoneurons in absence (control) or presence of the ryanodine receptor (RyR) antagonist, Dantrolene (Dantro., 50 µM, *n* = 5 mice, red) or the RyR agonist, caffeine (30 µM – 5 mM, *n* = 5 mice, red) or the SERCA pump blocker, thapsigargin (1 µM, *n* = 3 mice, red). Each circle represents an individual motoneuron. n.s., no significance (two-tailed Wilcoxon paired

test). Mean ± SEM. For detailed *P* values see Source Data. Source data are provided as a Source Data file.

# Supplementary Figure 7

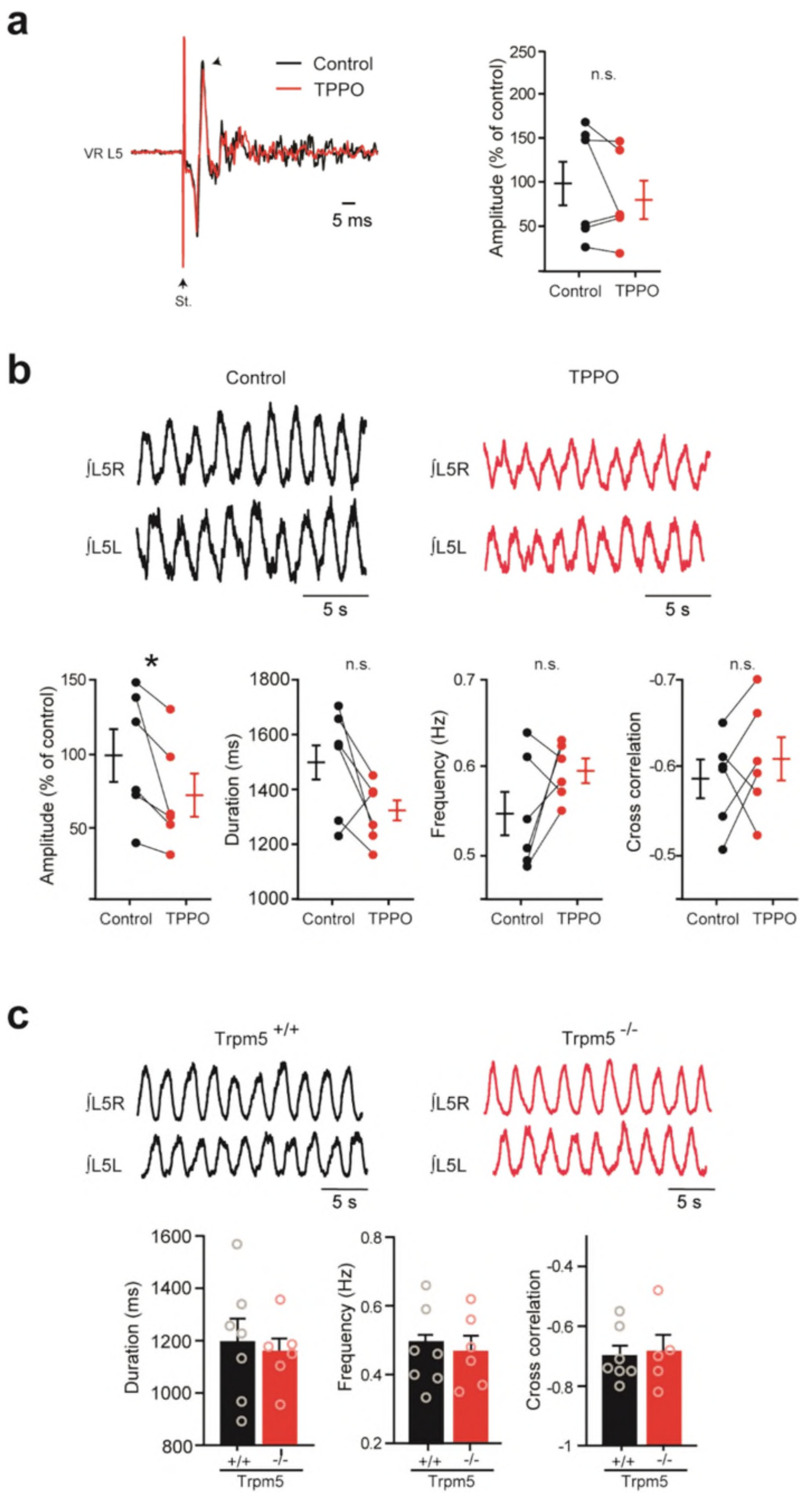

**Supplementary Fig. 7 (related to Fig. 6): Trpm5 channels regulate the gain of locomotor outputs without affecting either the glutamatergic synaptic transmission or the locomotor rhythm generation and coordination. a** Left: representative L5 ventral root activities (VR L5) evoked by a supramaximal stimulation (St.) of the ipsilateral dorsal root before (black) and after (red) bath-applying the Trpm5 blocker triphenylphosphine oxide, (TPPO, 30 µM). The arrowhead indicates the monosynaptic response, right: mean amplitude of the monosynaptic response relative to control values. **b,c** Top: Ventral-root recordings of NMA/5-HT-induced alternated rhythmic activity either recorded in isolated spinal cords from wild-type mice (*n* = 6) before (black) and after (red) bath-applying the Trpm5 blocker triphenylphosphine oxide, (TPPO, 30 µM; **b**) or derived for comparison between wild-type (black, *n* = 7 mice) and Trpm5$^{-/-}$ mice (red, *n* = 6 mice; **c**), bottom: Quantification of locomotor burst parameters. n.s., no significance; *$P$ < 0.05 (two-tailed Wilcoxon paired test for **a** and **b**; two-tailed Mann-Whitney test for **c**). Mean ± SEM. For detailed *P* values see Source Data. Source data are provided as a Source Data file.

# Supplementary Figure 8

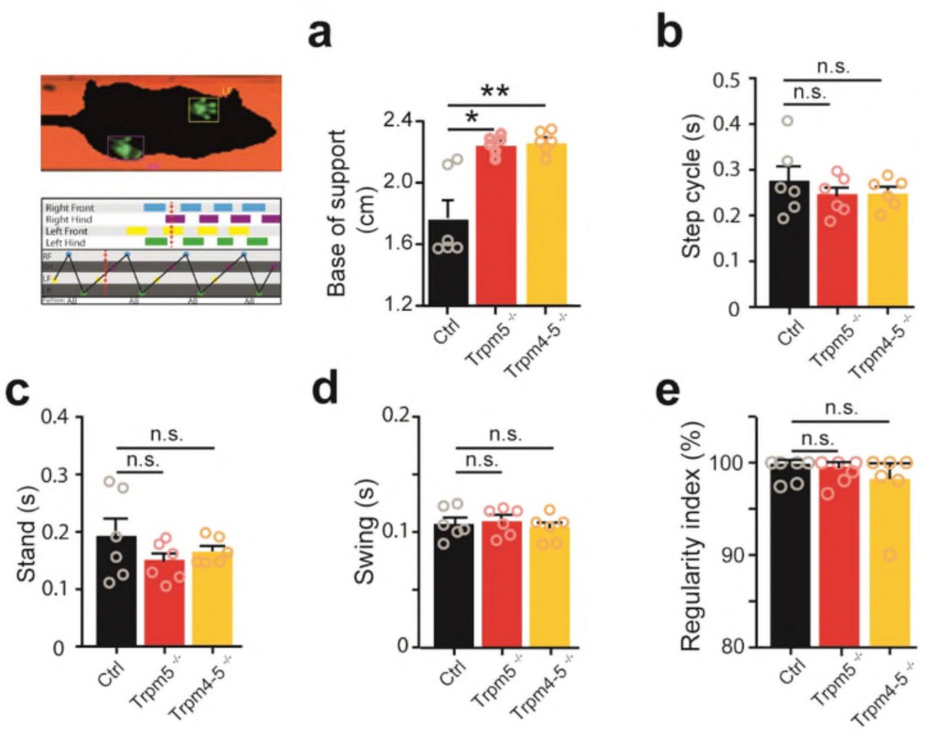


**Supplementary Fig. 8 (related to Fig. 7): Trpm5$^{-/-}$ mice display similar locomotor behaviors compared to double knockout Trpm4-5$^{-/-}$ mice. a-e** Base of support (**a**), step cycle (**b**), stand (**c**), swing (**d**) and mean regularity index (**e**) during CatWalk locomotion from 22-23 days old wild-type (black, *n* = 6 mice), Trpm5$^{-/-}$ (red, *n* = 6 mice) and Trpm4-5$^{-/-}$ (yellow, *n* = 6 mice) mice. n.s., no significance; $^{**}P < 0.01$ (one -way ANOVA). Mean ± SEM. For detailed *P* values see Source Data. Source data are provided as a Source Data file.

Supplementary Table 1

| | Control | | TRPM5 KO | | TRPM4-5 KO | | Ctrl-ShRNA | | TRPM5-ShRNA | |
|---|---|---|---|---|---|---|---|---|---|---|
| | Bistable (*n* = 40) | No Bistable (*n* = 4) | Bistable (*n* = 24) | No Bistable (*n* = 26) | Bistable (*n* = 10) | No Bistable (*n* = 10) | Bistable (*n* = 11) | No Bistable (*n* = 3) | Bistable (*n* = 5) | No Bistable (*n* = 11) |
| Area ($\mu m^2$) | 822.2 ± 40.9 | 669.0 ± 73.7 | 804.5 ± 36.6 | 803.3 ± 47.1 | 813.3 ± 79.9 | 718.7 ± 23.9 | 707.9 ± 47.2 | 721.7 ± 80.9 | 768.2 ± 81.1 | 761.0 ± 40.2 |
| Vrest (mV) | -76.13 ± 1.1 | -75.43 ± 3.4 | -77.2 ± 1.1 | -78.8 ± 1.5 | -76.2 ± 0.9 | -82.3 ± 1.9 | -75.04 ± 1.8 | -76.96 ± 3.8 | -79.5 ± 2.5 | -79.6 ± 1.3 |
| Rin (MΩ) | 21.7 ± 2.0 | 21.4 ± 2.3 | 29.4 ± 2.9 | 37.8 ± 4.7 | 38.1 ± 5.1 | 42.3 ± 4.5 | 24.2 ± 6.2 | 20.6 ± 6.4 | 20.6 ± 4.3 | 39.1 ± 3.4 |
| Rheobase (pA) | 1185 ± 88.0 | 1275 ± 232.3 | 827.1 ± 89.3 | 930.8 ± 172.0 | 795 ± 118.9 | 615 ± 48.3 | 1467 ± 285.7 | 1450 ± 284.4 | 1070 ± 313.3 | 327.3 ± 73.04 |

**Supplementary Table 1:** Morphological and electrophysiological properties of bistable and non-bistable motoneurons recorded from wild-type, $Trpm5^{-/-}$ and double knockout $Trpm4\text{-}5^{-/-}$ mice and wild-type mice transduced either with irrelevant shRNA or with a Trpm5-targeted shRNA. "*n*" indicates the numbers of cells. Data for control, Trpm5 KO, Trpm4-5 KO, Ctrl-ShRNA and Trpm5-ShRNA conditions were collected from 13, 5, 3, 3, and 4 mice, respectively. Mean ± SEM. Source data are provided as a Source Data file.
