# Supplementary material for: Trpm5 channels encode bistability of spinal motoneurons and ensure motor control of hindlimbs in mice
Source: Nat Commun. 2021 Nov 24;12:6815. doi: 10.1038/s41467-021-27113-x (PMC8613399; doi:10.1038/s41467-021-27113-x)
Supplement: Supplementary file 3 — Description of Additional Supplementary Files [file 41467_2021_27113_MOESM3_ESM.pdf]

Description of Additional Supplementary Files

File Name: Supplementary Movie 1

Description: Trpm5$^{-/-}$ mice display prolonged latency of the righting reflex compared to controls.

File Name: Supplementary Movie 2

Description: Trpm5$^{-/-}$ mice (P12) display a decreased swimming performance compared to controls.

File Name: Supplementary Movie 3

Description: Trpm5-ShRNA mice (P11) display strong deficits in the postural tone of hindlimbs compared to control-ShRNA.

File Name: Supplementary Movie 4

Description: Trpm5-ShRNA mice (P10) display strong deficits in the righting reflex compared to control-ShRNA.

File Name: Supplementary Movie 5

Description: Trpm5-ShRNA mice (P12) display a decreased swimming performance compared to control-ShRNA.
