# Supplementary material for: Trpm5 channels encode bistability of spinal motoneurons and ensure motor control of hindlimbs in mice
Source: Nat Commun. 2021 Nov 24;12:6815. doi: 10.1038/s41467-021-27113-x (PMC8613399; doi:10.1038/s41467-021-27113-x)
Supplement: Supplementary file 9 — Reporting Summary [file 41467_2021_27113_MOESM9_ESM.pdf]

nature research

Corresponding author(s): frederic brocard and remi bos

Last updated by author(s): October 20, 2021

Reporting Summary

Nature Research wishes to improve the reproducibility of the work that we publish. This form provides structure for consistency and transparency in reporting. For further information on Nature Research policies, see our Editorial Policies and the Editorial Policy Checklist.

Statistics

For all statistical analyses, confirm that the following items are present in the figure legend, table legend, main text, or Methods section.

n/a	Confirmed	
☐	☒	The exact sample size (*n*) for each experimental group/condition, given as a discrete number and unit of measurement
☐	☒	A statement on whether measurements were taken from distinct samples or whether the same sample was measured repeatedly
☐	☒	The statistical test(s) used AND whether they are one- or two-sided<br>*Only common tests should be described solely by name; describe more complex techniques in the Methods section.*
☒	☐	A description of all covariates tested
☐	☒	A description of any assumptions or corrections, such as tests of normality and adjustment for multiple comparisons
☐	☒	A full description of the statistical parameters including central tendency (e.g. means) or other basic estimates (e.g. regression coefficient) AND variation (e.g. standard deviation) or associated estimates of uncertainty (e.g. confidence intervals)
☐	☒	For null hypothesis testing, the test statistic (e.g. *F*, *t*, *r*) with confidence intervals, effect sizes, degrees of freedom and *P* value noted<br>*Give P values as exact values whenever suitable.*
☒	☐	For Bayesian analysis, information on the choice of priors and Markov chain Monte Carlo settings
☒	☐	For hierarchical and complex designs, identification of the appropriate level for tests and full reporting of outcomes
☒	☐	Estimates of effect sizes (e.g. Cohen's *d*, Pearson's *r*), indicating how they were calculated

Our web collection on statistics for biologists contains articles on many of the points above.

Software and code

Policy information about availability of computer code

Data collection	Clampex 10.3; Zen2.6-Zeiss; Catwalk xt10.6; EthoVision xt12; Neuron v7.7; Igor Pro v6.37; QuantStudioTM RealTime PCR Software v1.3; Quantity-One (Bio-Rad).
Data analysis	Clampfit 10.3; Graphpad Prism v7; Catwalk xt10.6; EthoVision xt12; Zen2.6 (Blue Edition) v2.6; Neuron v7.7; Igor Pro v6.37

For manuscripts utilizing custom algorithms or software that are central to the research but not yet described in published literature, software must be made available to editors and reviewers. We strongly encourage code deposition in a community repository (e.g. GitHub). See the Nature Research guidelines for submitting code & software for further information.

Data

Policy information about availability of data

All manuscripts must include a data availability statement. This statement should provide the following information, where applicable:

- Accession codes, unique identifiers, or web links for publicly available datasets
- A list of figures that have associated raw data
- A description of any restrictions on data availability

All data generated in this study that support our findings are presented within this paper, its supplementary materials, or in the source data. Accession codes will be made available upon reasonable request to the corresponding author.

Field-specific reporting

Please select the one below that is the best fit for your research. If you are not sure, read the appropriate sections before making your selection.

☒ Life sciences ☐ Behavioural & social sciences ☐ Ecological, evolutionary & environmental sciences

For a reference copy of the document with all sections, see nature.com/documents/nr-reporting-summary-flat.pdf

Life sciences study design

All studies must disclose on these points even when the disclosure is negative.

Sample size	No specific statistical methods were used to pre-determine sample size. The sample sizes were chosen based on available information on variability from previous behavioral, electrophysiological and immunohistochemical datasets (see refs 1,12,18,54) in compliance with ethical guidelines to minimize the number of animals used. The exact number of animal used in individual experiments are indicated in the figure legends.
Data exclusions	Electrophysiological data was only obtained if a stable patch was achieved. Briefly, to ensure optimum quality of intracellular recordings, membrane access resistance of whole-cell patched recording was monitored before and after recording and the data which with a >20% change was excluded from the analysis.
Replication	For electrophysiological data we recorded at least 6 neurons from at least two different animals. For in vivo behavioral tests we recorded at least 6 distinct animals in each group. All data were reproducible. All n are mentioned in figures.
Randomization	All samples and animals for the experiments were allocated randomly with similar number in each group.
Blinding	Investigators were blinded to the genotypes of the samples or to the treatment of experimental subjects (Trpm5 shRNA or scrambled treated). Western Blots were not blinded to group the samples in desired order. Data was analyzed blind to condition.

Reporting for specific materials, systems and methods

We require information from authors about some types of materials, experimental systems and methods used in many studies. Here, indicate whether each material, system or method listed is relevant to your study. If you are not sure if a list item applies to your research, read the appropriate section before selecting a response.

Materials & experimental systems

n/a	Involved in the study	
☐	☒	Antibodies
☐	☒	Eukaryotic cell lines
☒	☐	Palaeontology and archaeology
☐	☒	Animals and other organisms
☒	☐	Human research participants
☒	☐	Clinical data
☒	☐	Dual use research of concern

Methods

n/a	Involved in the study	
☒	☐	ChIP-seq
☒	☐	Flow cytometry
☒	☐	MRI-based neuroimaging

Antibodies

Antibodies used	Antibodies used include: Goat polyclonal Choline Acetyltransferase antibody (Millipore, Cat# AB144P); Rabbit polyclonal Glial Fibrillary Acidic Protein (GFAP) antibody (AgilentDako, Cat# Z0334); Donkey polyclonal anti-goat gamma immunoglobins antibody, AlexaFluor Plus 555 (Thermo Fisher, Cat# A32816); Rabbit polyclonal Trpm5 antibody (Alomone, Cat# ACC-045); Goat polyclonal anti-rabbit gamma immunoglobins antibody, Horseradish Peroxidase (Thermo Fisher, Cat# 31460).
Validation	All the antibodies are commercially available; representative blots are shown in their data-sheet as well as background references.<br>Goat polyclonal Choline Acetyltransferase antibody: https://www.merckmillipore.com/FR/fr/product/Anti-Choline-Acetyltransferase-Antibody,MM_NF-AB144P.<br>Rabbit polyclonal Glial Fibrillary Acidic Protein antibody (AgilentDako, Cat# Z0334): https://www.agilent.com/en/product/immunohistochemistry/antibodies-controls/primary-antibodies/glial-fibrillary-acidic-protein-(concentrate)-76683<br>AlexaFluor Plus 555 donkey polyclonal anti-goat gamma immunoglobins antibody: https://www.thermofisher.com/antibody/product/Donkey-anti-Goat-IgG-H-L-Highly-Cross-Adsorbed-Secondary-Antibody-Polyclonal/A32816<br>Rabbit polyclonal Trpm5 antibody: https://www.alomone.com/p/anti-trpm5/ACC-045<br>Goat polyclonal anti-rabbit gamma immunoglobins antibody, Horseradish Peroxidase : https://www.thermofisher.com/antibody/product/Goat-anti-Rabbit-IgG-H-L-Secondary-Antibody-Polyclonal/31460

Eukaryotic cell lines

Policy information about cell lines

Cell line source(s)	Human Embryonic Kidney 293
Authentication	None of the cell lines have been authenticated.
Mycoplasma contamination	Cell lines were not tested for mycoplasma contamination but no indication of contamination was observed.
Commonly misidentified lines (See ICLAC register)	No commonly misidentified cell lines were used.

Animals and other organisms

Policy information about studies involving animals; ARRIVE guidelines recommended for reporting animal research

Laboratory animals	Non-transgenic mice C57/Bl6 female and male 1 day-old to 5-weeks-old; Transgenic mice Trpv1-3-/- and Trpv2-/- with C57/Bl6 background female and male 5 to 12-days-old; Transgenic mice Trpm4-/- and Trpm5-/- with C57/Bl6 background female and male 1 day-old to 5-weeks-old. Room temperature was kept between 21-24°C and between 40-60% relative humidity.
Wild animals	Study did not involve wild animals
Field-collected samples	Study did not involve samples collected from the field
Ethics oversight	All animal care and use were conformed to the French regulations (Décret 2010-118) and approved by the local ethics committee (Comité d'Ethique en Neurosciences INT-Marseille, CE71 Nb A1301404, authorization Nb 2018110819197361).

Note that full information on the approval of the study protocol must also be provided in the manuscript.
